# Fundamental Solutions to Fractional Heat Conduction in Two Joint Half-Lines Under Conditions of Nonperfect Thermal Contact

**DOI:** 10.3390/e27090965

**Published:** 2025-09-16

**Authors:** Yuriy Povstenko, Tamara Kyrylych, Viktor Dashkiiev, Andrzej Yatsko

**Affiliations:** 1 Department of Mathematics and Computer Sciences, Faculty of Science and Technology, Jan Dlugosz University in Czestochowa, al. Armii Krajowej 13/15, 42-200 Czestochowa, Poland; t.kyrylych@ujd.edu.pl; 2 Faculty of Mechanical Engineering, Cracow University of Technology, 37 Jana Pawła II Av., 31-864 Cracow, Poland; viktor.dashkiiev@pk.edu.pl; 3 Department of Mathematics, Faculty of Civil Engineering, Environmental and Geodesic Sciences, Koszalin University of Technology, Sniadeckich 2, 75-453 Koszalin, Poland; andrzej.jacko@gmail.com

**Keywords:** fractional calculus, fractional heat conduction, fractional diffusion, Caputo derivative, Mittag-Leffler function, nonperfect thermal contact

## Abstract

At the interface dividing two media, an area appears that has its own physical characteristics which differ from the properties of the bulk materials. The small width of the interface area permits considering this area as a two-dimensional median surface with the specified physical characteristics. The fundamental solutions to the Cauchy problem as well as to the source problem are considered for fractional heat conduction in two joint half-lines under conditions of nonperfect thermal contact. The specific example of classical heat conduction is also investigated.

## 1. Introduction

The usual heat conduction, based on the standard Fourier law, is in many cases quite acceptable. Nevertheless, in media with complex internal structure (porous, granular, random, and fractal materials, glasses, polymers, semiconductors, living tissues, etc.) the classical approach is insufficient. In these instances, fractional calculus is an excellent mathematical tool for the description of complicated physical processes [1,2,3,4,5,6,7,8,9,10].

At the interface dividing two media, an area appears that has its own physical characteristics which differ from the properties of the bulk materials. The small width of the interface area permits considering this area as a two-dimensional median surface with the specified physical characteristics (reduced heat capacity, reduced thermal conductivity, and reduced thermal resistance). This approach results in the conditions of nonperfect thermal contact between two media. There is a voluminous literature on reducing the three-dimensional heat conduction problem to the two-dimensional analog, both for the standard Fourier law and for more general nonclassical theories. The interested reader is referred, for example, to the discussion of the methods of such reducing in [9,11].

Recall the generalized boundary conditions of nonperfect thermal contact for time-fractional heat conduction. Consider a body composed of three parts marked by the indices 1, 2, and 0 for two contacting areas and the interfacial region between them, respectively. Heat conduction in each domain is described by the time-fractional equation:

(1) $$\begin{array}{ccc} C_{1}\;\frac{\partial^{\;\alpha }T_{1}}{\partial t^{\alpha }} & = & k_{1}\Delta \;T_{1}, \end{array}$$

(2) $$\begin{array}{ccc} C_{2}\;\frac{\partial^{\;\beta }T_{2}}{\partial t^{\beta }} & = & k_{2}\Delta \;T_{2}, \end{array}$$

(3) $$\begin{array}{ccc} C_{0}\;\frac{\partial^{\;\gamma }T_{0}}{\partial t^{\gamma }} & = & k_{0}\Delta \;T_{0}. \end{array}$$

Here, *C* denotes the heat capacity and *k* is the thermal conductivity coefficient. The operators of fractional differentiation are explained in Appendix A.

Assuming perfect thermal contact conditions at the boundaries between the contact bodies and the intermediate region and taking into account a small constant width *h* of the intermediate layer, the following boundary conditions of nonperfect thermal contact can be achieved at the median surface [9,11]:

(4) $$\begin{array}{ccc} C_{\Sigma }\;\frac{\partial^{\gamma }\left(T_{1}+T_{2}\right)}{\partial t^{\gamma }} & = & k_{\Sigma }\;\Delta_{\Sigma }\left(T_{1}+T_{2}\right)\\ & + & 2\left(k_{1}D_{RL}^{\gamma -\alpha }\frac{\partial T_{1}}{\partial z}{|}_{z=0}-k_{2}D_{RL}^{\gamma -\beta }\frac{\partial T_{2}}{\partial z}{|}_{z=0}\right), \end{array}$$

(5) $$\begin{array}{ccc} & \;\;\; & \;\;\;\;\;\;\;\;\;\;\;\;\;\;\;\;\;C_{\Sigma }\;\frac{\partial^{\gamma }\left(T_{1}-T_{2}\right)}{\partial t^{\gamma }}=k_{\Sigma }\;\Delta_{\Sigma }\left(T_{1}-T_{2}\right)\\ & + & 6\left(k_{1}D_{RL}^{\gamma -\alpha }\frac{\partial T_{1}}{\partial z}{|}_{z=0}+k_{2}D_{RL}^{\gamma -\beta }\frac{\partial T_{2}}{\partial z}{|}_{z=0}\right)-\frac{12}{R_{\Sigma }}\;\left(T_{1}-T_{2}\right), \end{array}$$

where $\Delta_{\Sigma }$ is the surface Laplace operator, *z* is the coordinate normal to the median surface, $C_{\Sigma }=2hC_{0}$ denotes the reduced heat capacity, $k_{\Sigma }=2hk_{0}$ means the reduced thermal conductivity coefficient, and $R_{\Sigma }=k_{0}/\left(2h\right)$ stands for the reduced thermal resistance of the median surface.

In the instance of classical heat conduction when α=β=γ=1, Equations (4) and (5) lead to the conditions of nonperfect contact obtained by Podstrigach [12]. When the reduced thermal properties of the median surface are equal to zero, Conditions (4) and (5) give the perfect thermal contact conditions for describing fractional heat conduction [9,13].

In the present paper, we study the fundamental solutions to the Cauchy problem as well as to the source problem for fractional heat conduction in two joint half-lines under conditions of nonperfect thermal contact. The specific example of classical heat conduction is also investigated.

## 2. Fundamental Solution to the Cauchy Problem

For problems with one spatial coordinate, the surface Laplace operator $\Delta_{\Sigma }$ equals zero, as the contact plane is normal to the *x*-axis. Hereinafter, we restrict ourselves to the particular case when α=β=γ, $R_{\Sigma }=0$. Hence, we have the time-fractional heat conduction equations

(6) $$\frac{\partial^{\alpha }T_{1}\left(x,t\right)}{\partial t^{\alpha }}=a_{1}\;\frac{\partial^{2}T_{1}\left(x,t\right)}{\partial x^{2}},\;x>0,\;0<\alpha \leq 2,$$

(7) $$\frac{\partial^{\alpha }T_{2}\left(x,t\right)}{\partial t^{\alpha }}=a_{2}\;\frac{\partial^{2}T_{2}\left(x,t\right)}{\partial x^{2}},\;x<0,\;0<\alpha \leq 2,$$

under the initial conditions

(8) $$\begin{array}{ccc} t=0: & \;T_{1}\left(x,t\right)=p_{0}\;\delta \left(x-l\right), & \;\;x>0,\;\;\;0<\alpha \leq 2, \end{array}$$

(9) $$\begin{array}{ccc} t=0: & \;\frac{\partial T_{1}\left(x,t\right)}{\partial t}=0, & \;\;x>0,\;\;\;1<\alpha \leq 2, \end{array}$$

(10) $$\begin{array}{ccc} t=0: & \;T_{2}\left(x,t\right)=0, & \;\;x<0,\;\;\;0<\alpha \leq 2, \end{array}$$

(11) $$\begin{array}{ccc} t=0: & \;\frac{\partial T_{2}\left(x,t\right)}{\partial t}=0, & \;\;x<0,\;\;\;1<\alpha \leq 2, \end{array}$$

and under the boundary conditions

(12) $$x=0:\;\;T_{1}\left(x,t\right)=T_{2}\left(x,t\right),$$

(13) $$x=0:\;\;C_{\Sigma }\;\frac{\partial^{\alpha }\left[T_{1}\left(x,t\right)+T_{2}\left(x,t\right)\right]}{\partial t^{\alpha }}=2\left[k_{1}\frac{\partial T_{1}\left(x,t\right)}{\partial x}-k_{2}\frac{\partial T_{2}\left(x,t\right)}{\partial x}\right].$$

The conditions at infinity are also adopted:

(14) $$\lim_{x\to \infty }T_{1}\left(x,t\right)=0,\;\lim_{x\to -\infty }T_{2}\left(x,t\right)=0.$$

The Laplace integral transform with respect to time *t* leads to the following equations:

(15) $$\begin{array}{ccc} s^{\alpha }T_{1}^{*}-s^{\alpha -1}p_{0}\;\delta \left(x-l\right)=a_{1}\;\frac{\partial^{2}T_{1}^{*}}{\partial x^{2}}, & \; & x>0,\;0<\alpha \leq 2, \end{array}$$

(16) $$\begin{array}{ccc} \;s^{\alpha }T_{2}^{*}=a_{2}\;\frac{\partial^{2}T_{2}^{*}}{\partial x^{2}}, & \; & x<0,\;0<\alpha \leq 2, \end{array}$$

and the corresponding boundary conditions

(17) $$\begin{array}{ccc} x=0: & \; & T_{1}^{*}=T_{2}^{*}, \end{array}$$

(18) $$\begin{array}{ccc} x=0: & \; & C_{\Sigma }\;s^{\alpha }\left(T_{1}^{*}+T_{2}^{*}\right)=2\left(k_{1}\frac{\partial T_{1}^{*}}{\partial x}-k_{2}\frac{\partial T_{2}^{*}}{\partial x}\right). \end{array}$$

At this stage, we introduce two unknown functions:

(19) $$\frac{\partial T_{1}^{*}}{\partial x}{|}_{x=0}=\varphi^{*},\;\frac{\partial T_{2}^{*}}{\partial x}{|}_{x=0}=\psi^{*}.$$

The Fourier cosine transforms with respect to the spatial coordinate *x* in the domains 0≤x<∞ and −∞<x≤0 give

(20) $$\begin{array}{ccc} {\tilde{T}}_{1}^{*} & = & \frac{p_{0}s^{\alpha -1}}{s^{\alpha }+a_{1}\xi^{2}}\;\cos\left(l\xi \right)-a_{1}\varphi^{*}\;\frac{1}{s^{\alpha }+a_{1}\xi^{2}}, \end{array}$$

(21) $$\begin{array}{ccc} {\tilde{T}}_{2}^{*} & = & a_{2}\psi^{*}\;\frac{1}{s^{\alpha }+a_{2}\xi^{2}}. \end{array}$$

Here, the tilde denotes the Fourier transform; ξ is the transform variable.

The inverse Fourier cosine transforms result in

(22) $$\begin{array}{ccc} T_{1}^{*} & = & \frac{2}{\pi }\int_{0}^{\infty }\frac{p_{0}s^{\alpha -1}}{s^{\alpha }+a_{1}\xi^{2}}\;\cos\left(l\xi \right)\;\cos\left(x\xi \right)\;d\xi \\ & - & \frac{2}{\pi }\int_{0}^{\infty }\frac{a_{1}\varphi^{*}}{s^{\alpha }+a_{1}\xi^{2}}\;\cos\left(x\xi \right)\;d\xi ,\;x>0, \end{array}$$

(23) $$\begin{array}{ccc} T_{2}^{*} & = & \frac{2}{\pi }\int_{-\infty }^{0}\frac{a_{2}\psi^{*}}{s^{\alpha }+a_{2}\xi^{2}}\;\cos\left(x\xi \right)\;d\xi ,\;x<0, \end{array}$$

and, after taking into account that [14]

(24) $$\int_{0}^{\infty }\frac{1}{\xi^{2}+c^{2}}\;\cos\left(b\xi \right)\;d\xi =\frac{\pi }{2c}\;e^{-bc},\;b>0,\;c>0,$$

we arrive at the expressions for temperature in the Laplace transform domain:

(25) $$\begin{array}{ccc} T_{1}^{*} & = & \frac{p_{0}}{2\sqrt{a_{1}}}\;s^{\alpha /2-1}\left[\exp\left(-\frac{x+l}{\sqrt{a_{1}}}\;s^{\alpha /2}\right)+\exp\left(-\frac{\left|x-l\right|}{\sqrt{a_{1}}}\;s^{\alpha /2}\right)\right]\\ & - & \varphi^{*}\frac{\sqrt{a_{1}}}{s^{\alpha /2}}\;\exp\left(-\frac{x}{\sqrt{a_{1}}}\;s^{\alpha /2}\right),\;x>0, \end{array}$$

(26) $$\begin{array}{ccc} T_{2}^{*} & = & \psi^{*}\frac{\sqrt{a_{2}}}{s^{\alpha /2}}\;\exp\left(-\frac{\left|x\right|}{\sqrt{a_{2}}}\;s^{\alpha /2}\right),\;x<0. \end{array}$$

It follows from Equations (25) and (26) that, at the boundary,

(27) $$\begin{array}{ccc} x=0: & \; & T_{1}^{*}=\frac{p_{0}}{\sqrt{a_{1}}}\;s^{\alpha /2-1}\exp\left(-\frac{l}{\sqrt{a_{1}}}\;s^{\alpha /2}\right)-\varphi^{*}\frac{\sqrt{a_{1}}}{s^{\alpha /2}}, \end{array}$$

(28) $$\begin{array}{ccc} x=0: & \; & T_{2}^{*}=\psi^{*}\frac{\sqrt{a_{2}}}{s^{\alpha /2}}, \end{array}$$

and, from Conditions (17) and (18), we obtain a system of linear equations for determining two unknown functions $\varphi^{*}$ and $\psi^{*}$:

(29) $$\begin{array}{ccc} & & \frac{p_{0}}{\sqrt{a_{1}}}\;s^{\alpha /2-1}\exp\left(-\frac{l}{\sqrt{a_{1}}}\;s^{\alpha /2}\right)-\varphi^{*}\frac{\sqrt{a_{1}}}{s^{\alpha /2}}=\psi^{*}\frac{\sqrt{a_{2}}}{s^{\alpha /2}}, \end{array}$$

(30) $$\begin{array}{ccc} & & C_{\Sigma }\;s^{\alpha /2}\sqrt{a_{2}}\;\psi^{*}=k_{1}\varphi^{*}-k_{2}\psi^{*}, \end{array}$$

having the solution

(31) $$\begin{array}{ccc} \psi^{*} & = & \frac{p_{0}k_{1}s^{\alpha -1}}{\sqrt{a_{1}}\left(k_{1}\sqrt{a_{2}}+k_{2}\sqrt{a_{1}}+C_{\Sigma }\;\sqrt{a_{1}a_{2}}s^{\alpha /2}\right)}\;\exp\left(-\frac{l}{\sqrt{a_{1}}}\;s^{\alpha /2}\right), \end{array}$$

(32) $$\begin{array}{ccc} \varphi^{*} & = & \frac{C_{\Sigma }\;\sqrt{a_{2}}s^{\alpha /2}+k_{2}}{k_{1}}\;\psi^{*}. \end{array}$$

From Equations (25), (26), (31), and (32), we obtain

(33) $$\begin{array}{ccc} T_{1}^{*} & = & \frac{p_{0}}{2\sqrt{a_{1}}}\;s^{\alpha /2-1}\left[\exp\left(-\frac{\left|x-l\right|}{\sqrt{a_{1}}}\;s^{\alpha /2}\right)-\exp\left(-\frac{x+l}{\sqrt{a_{1}}}\;s^{\alpha /2}\right)\right]\\ & + & \frac{p_{0}k_{1}}{a_{1}C_{\Sigma }}\;s^{\alpha /2-1}\exp\left(-\frac{x+l}{\sqrt{a_{1}}}\;s^{\alpha /2}\right)\frac{1}{s^{\alpha /2}+b},\;x>0, \end{array}$$

(34) $$\begin{array}{ccc} T_{2}^{*} & = & \frac{p_{0}k_{1}}{a_{1}C_{\Sigma }}\;s^{\alpha /2-1}\exp\left[-\left(\frac{\left|x\right|}{\sqrt{a_{2}}}+\frac{l}{\sqrt{a_{1}}}\right)s^{\alpha /2}\right]\frac{1}{s^{\alpha /2}+b},\;x<0, \end{array}$$

where

(35) $$b=\frac{k_{1}\sqrt{a_{2}}+k_{2}\sqrt{a_{1}}}{\sqrt{a_{1}a_{2}}\;C_{\Sigma }}.$$

Taking into account the formulae for the inverse Laplace transform [15,16,17]

(36) $$L^{-1}\left\{\frac{s^{\alpha -\beta }}{s^{\alpha }+b}\right\}=t^{\beta -1}\;E_{\alpha ,\beta }\left(-bt^{\alpha }\right),$$

(37) $$L^{-1}\left\{s^{\alpha -1}\exp\left(-\lambda s^{\alpha }\right)\right\}=\frac{1}{t^{\alpha }}M\left(\alpha ;\frac{\lambda }{t^{\alpha }}\right),\;0<\alpha <1,\;\;\;\;\lambda >0,$$

and using the convolution theorem, we finally obtain

(38) $$\begin{array}{ccc} T_{1}\left(x,t\right) & = & \frac{p_{0}}{2\sqrt{a_{1}}t^{\alpha /2}}\left[M\left(\frac{\alpha }{2};\frac{\left|x-l\right|}{\sqrt{a_{1}}t^{\alpha /2}}\right)-M\left(\frac{\alpha }{2};\frac{x+l}{\sqrt{a_{1}}t^{\alpha /2}}\right)\right]\\ & + & \frac{p_{0}k_{1}}{a_{1}C_{\Sigma }}\int_{0}^{t}\frac{{\left(t-\tau \right)}^{\alpha /2-1}}{\tau^{\alpha /2}}\;M\left(\frac{\alpha }{2};\frac{x+l}{\sqrt{a_{1}}\tau^{\alpha /2}}\right)\\ & \times & E_{\alpha /2,\alpha /2}\left[-b{\left(t-\tau \right)}^{\alpha /2}\right]d\tau ,\;x>0, \end{array}$$

(39) $$\begin{array}{ccc} T_{2}\left(x,t\right) & = & \frac{p_{0}k_{1}}{a_{1}C_{\Sigma }}\int_{0}^{t}\frac{{\left(t-\tau \right)}^{\alpha /2-1}}{\tau^{\alpha /2}}\;M\left[\frac{\alpha }{2};\left(\frac{\left|x\right|}{\sqrt{a_{2}}}+\frac{l}{\sqrt{a_{1}}}\right)\frac{1}{\tau^{\alpha /2}}\right]\\ & \times & E_{\alpha /2,\alpha /2}\left[-b{\left(t-\tau \right)}^{\alpha /2}\right]d\tau ,\;x<0. \end{array}$$

Here, $E_{\alpha ,\beta }$ is the Mittag-Leffler-type function in two parameters [17,18]

(40) $$E_{\alpha ,\beta }\left(z\right)=\sum_{k=0}^{\infty }\frac{z^{k}}{\Gamma (\alpha k+\beta )},\;\alpha >0,\;\;\;\;\beta >0,\;\;\;\;z\in C,$$

and the Mainardi function M(α;z) is introduced by the series representation [15,16,17]

(41) $$M\left(\alpha ;z\right)=\sum_{k=0}^{\infty }\frac{{\left(-1\right)}^{k}z^{k}}{k!\;\Gamma [-\alpha k+(1-\alpha )]},\;0<\alpha <1,\;\;z\in C.$$

Below, we present two particular cases of the obtained solution.

Classical heat conduction (α=1):

(42) $$\begin{array}{ccc} T_{1}\left(x,t\right) & \;\;= & \;\;\frac{p_{0}}{2\sqrt{\pi a_{1}t}}\left\{\exp\left[-\frac{{\left(x-l\right)}^{2}}{4a_{1}t}\right]-\exp\left[-\frac{{\left(x+l\right)}^{2}}{4a_{1}t}\right]\right\}\\ & \;\;+ & \;\;\frac{p_{0}k_{1}}{a_{1}C_{\Sigma }}\;\exp\left(\frac{x+l}{\sqrt{a_{1}}}\;b+b^{2}t\right)\mathrm{erfc}\left(\frac{x+l}{2\sqrt{a_{1}t}}+b\sqrt{t}\right),\;x>0, \end{array}$$

(43) $$\begin{array}{ccc} T_{2}\left(x,t\right) & \;\;= & \;\;\frac{p_{0}k_{1}}{a_{1}C_{\Sigma }}\;\exp\left[\left(\frac{\left|x\right|}{\sqrt{a_{2}}}+\frac{l}{\sqrt{a_{1}}}\right)b+b^{2}t\right)\\ & \;\;\times & \;\;\mathrm{erfc}\left(\frac{\left|x\right|}{2\sqrt{a_{2}t}}+\frac{l}{2\sqrt{a_{1}t}}+b\sqrt{t}\right),\;\;x<0. \end{array}$$

Ballistic heat conduction (α=2):

(44) $$\begin{array}{ccc} T_{1}\left(x,t\right) & \;\;= & \;\;\frac{p_{0}}{2}\left[\delta \left(\sqrt{a_{1}}t-\left|x-l\right|\right)-\delta \left(\sqrt{a_{1}}t-x-l\right)\right]\\ & \;\;+ & \;\;\frac{p_{0}k_{1}}{a_{1}C_{\Sigma }}\;\exp\left[-b\left(t-\frac{x+l}{\sqrt{a_{1}}}\right)\right]H\left(t-\frac{x+l}{\sqrt{a_{1}}}\right),\;x>0, \end{array}$$

(45) $$\begin{array}{ccc} T_{2}\left(x,t\right) & \;\;= & \;\;\frac{p_{0}k_{1}}{a_{1}C_{\Sigma }}\;\exp\left[-b\left(t-\frac{\left|x\right|}{\sqrt{a_{2}}}-\frac{l}{\sqrt{a_{1}}}\right)\right]\\ & \;\;\times & \;\;H\left(t-\frac{\left|x\right|}{\sqrt{a_{2}}}-\frac{l}{\sqrt{a_{1}}}\right),\;x<0, \end{array}$$

where H(x) is the Heaviside step function.

**Figure 1:**
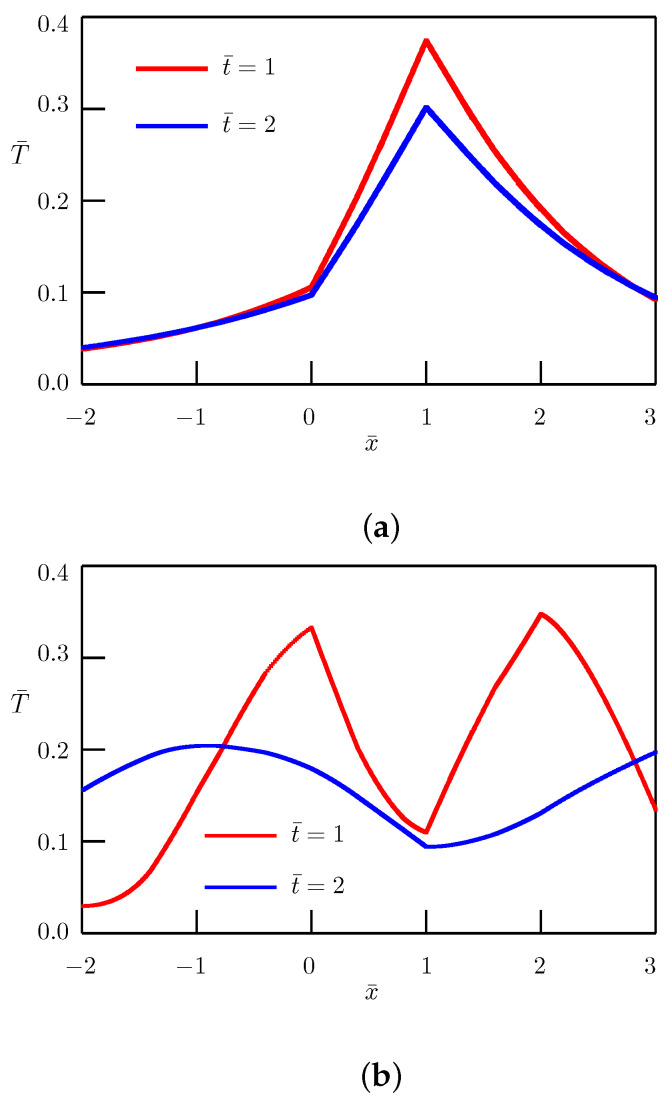
Fundamental solution to the Cauchy problem. Dependence of temperature on distance for α=0.5—(**a**) and α=1.5—(**b**).

The dependence of temperature on distance is depicted in Figure 1 for typical values of the order of fractional derivative (0<α<1 in Figure 1a and 1<α<2 in Figure 1b). In numerical simulation, we have utilized the following nondimensional quantities:

(46) $$\begin{array}{ccc} & & {\bar{T}}_{1}=\frac{l}{p_{0}}T_{1},\;{\bar{T}}_{2}=\frac{l}{p_{0}}T_{2},\;\bar{x}=\frac{x}{l},\;\bar{t}=\frac{a_{1}^{1/\alpha }}{l^{2/\alpha }}\;t,\\ & & \beta =\frac{k_{1}l}{a_{1}C_{\Sigma }},\;\gamma =\frac{k_{1}}{k_{2}},\;\epsilon =\sqrt{\frac{a_{1}}{a_{2}}}, \end{array}$$

and have taken the values β=2,γ=2,ϵ=0.75.

## 3. Fundamental Solution to the Source Problem

We solve the time-fractional heat conduction equations

(47) $$\frac{\partial^{\alpha }T_{1}\left(x,t\right)}{\partial t^{\alpha }}=a_{1}\;\frac{\partial^{2}T_{1}\left(x,t\right)}{\partial x^{2}}+q_{0}\delta \left(x-l\right)\;\delta \left(t\right),\;x>0,\;0<\alpha \leq 2,$$

(48) $$\frac{\partial^{\alpha }T_{2}\left(x,t\right)}{\partial t^{\alpha }}=a_{2}\;\frac{\partial^{2}T_{2}\left(x,t\right)}{\partial x^{2}},\;x<0,\;0<\alpha \leq 2,$$

under the zero initial conditions

(49) $$\begin{array}{ccc} t=0: & \;T_{1}\left(x,t\right)=0, & \;\;x>0,\;\;\;0<\alpha \leq 2, \end{array}$$

(50) $$\begin{array}{ccc} t=0: & \;\frac{\partial T_{1}\left(x,t\right)}{\partial t}=0, & \;\;x>0,\;\;\;1<\alpha \leq 2, \end{array}$$

(51) $$\begin{array}{ccc} t=0: & \;T_{2}\left(x,t\right)=0, & \;\;x<0,\;\;\;0<\alpha \leq 2, \end{array}$$

(52) $$\begin{array}{ccc} t=0: & \;\frac{\partial T_{2}\left(x,t\right)}{\partial t}=0, & \;\;x<0,\;\;\;1<\alpha \leq 2, \end{array}$$

and under the boundary conditions of nonperfect thermal contact in (12) and (13).

The Laplace integral transform with respet to time *t* and the Fourier cosine transforms with respect to the spatial coordinates *x* result in the expressions for temperature in the Laplace transform domain:

(53) $$\begin{array}{ccc} T_{1}^{*} & = & \frac{q_{0}}{2\sqrt{a_{1}}}\;s^{-\alpha /2}\left[\exp\left(-\frac{\left|x-l\right|}{\sqrt{a_{1}}}\;s^{\alpha /2}\right)-\exp\left(-\frac{x+l}{\sqrt{a_{1}}}\;s^{\alpha /2}\right)\right]\\ & + & \frac{q_{0}k_{1}}{a_{1}C_{\Sigma }}\;s^{-\alpha /2}\exp\left(-\frac{x+l}{\sqrt{a_{1}}}\;s^{\alpha /2}\right)\frac{1}{s^{\alpha /2}+b},\;x>0, \end{array}$$

(54) $$T_{2}^{*}=\frac{q_{0}k_{1}}{a_{1}C_{\Sigma }}\;s^{-\alpha /2}\exp\left[-\left(\frac{\left|x\right|}{\sqrt{a_{2}}}+\frac{l}{\sqrt{a_{1}}}\right)s^{\alpha /2}\right]\frac{1}{s^{\alpha /2}+b},\;x<0.$$

Now, we use Equation (36) and the following equation for the inverse Laplace transform: [15,16]

(55) $$L^{-1}\left\{s^{-\beta }\exp\left(-\lambda s^{\alpha }\right)\right\}=t^{\beta -1}W\left(-\alpha ,\beta ;-\lambda t^{-\alpha }\right),\;\;\;0<\alpha <1,\;\;\;\lambda >0,$$

where the Wright function $W\left(-\alpha ,\beta ;z\right)$ is expressed as [15,16,17,18,19]

(56) $$W\left(\alpha ,\beta ;z\right)=\sum_{k=0}^{\infty }\frac{z^{k}}{k!\;\Gamma (\alpha k+\beta )},\;\alpha >-1,\;z\in C.$$

In such a way, we arrive at the solution

(57) $$\begin{array}{ccc} T_{1}\left(x,t\right) & = & \frac{q_{0}}{2\sqrt{a_{1}}}t^{\alpha /2-1}[W\left(-\frac{\alpha }{2},\frac{\alpha }{2};-\frac{\left|x-l\right|}{\sqrt{a_{1}}t^{\alpha /2}}\right)\\ & - & W\left(-\frac{\alpha }{2},\frac{\alpha }{2};-\frac{x+l}{\sqrt{a_{1}}t^{\alpha /2}}\right)]\\ & + & \frac{q_{0}k_{1}}{a_{1}C_{\Sigma }}\int_{0}^{t}\tau^{\alpha /2-1}\;W\left(-\frac{\alpha }{2},\frac{\alpha }{2};-\frac{x+l}{\sqrt{a_{1}}\tau^{\alpha /2}}\right)\\ & \times & E_{\alpha /2,\alpha /2}\left[-b{\left(t-\tau \right)}^{\alpha /2}\right]{\left(t-\tau \right)}^{\alpha /2-1}d\tau ,\;x>0, \end{array}$$

(58) $$\begin{array}{ccc} T_{2}\left(x,t\right) & = & \frac{q_{0}k_{1}}{a_{1}C_{\Sigma }}\int_{0}^{t}\tau^{\alpha /2-1}\;W\left[-\frac{\alpha }{2},\frac{\alpha }{2};-\left(\frac{\left|x\right|}{\sqrt{a_{2}}}+\frac{l}{\sqrt{a_{1}}}\right)\frac{1}{\tau^{\alpha /2}}\right]\\ & \times & E_{\alpha /2,\alpha /2}\left[-b{\left(t-\tau \right)}^{\alpha /2}\right]{\left(t-\tau \right)}^{\alpha /2-1}d\tau ,\;x<0. \end{array}$$

In the case of ballistic heat conduction (α=2), we have

(59) $$\begin{array}{ccc} T_{1}\left(x,t\right) & \;\;= & \;\;\frac{q_{0}}{2\sqrt{a_{1}}}H\left(t-\frac{\left|x-l\right|}{\sqrt{a_{1}}}\right)\\ & \;\;- & \;\;\left\{\frac{q_{0}}{2\sqrt{a_{1}}}-\frac{q_{0}k_{1}}{a_{1}C_{\Sigma }\;b}+\frac{q_{0}k_{1}}{a_{1}C_{\Sigma }\;b}\;\exp\left[-b\left(t-\frac{x+l}{\sqrt{a_{1}}}\right)\right]\right\}\\ & \;\;\times & \;\;H\left(t-\frac{x+l}{\sqrt{a_{1}}}\right),\;x>0, \end{array}$$

(60) $$\begin{array}{ccc} T_{2}\left(x,t\right) & \;\;= & \;\;\frac{q_{0}k_{1}}{a_{1}C_{\Sigma }\;b}\left\{1-\exp\left[-b\left(t-\frac{\left|x\right|}{\sqrt{a_{2}}}-\frac{l}{\sqrt{a_{1}}}\right)\right]\right\}\\ & \;\;\times & \;\;H\left(t-\frac{\left|x\right|}{\sqrt{a_{2}}}-\frac{l}{\sqrt{a_{1}}}\right),\;x<0. \end{array}$$

**Figure 2:**
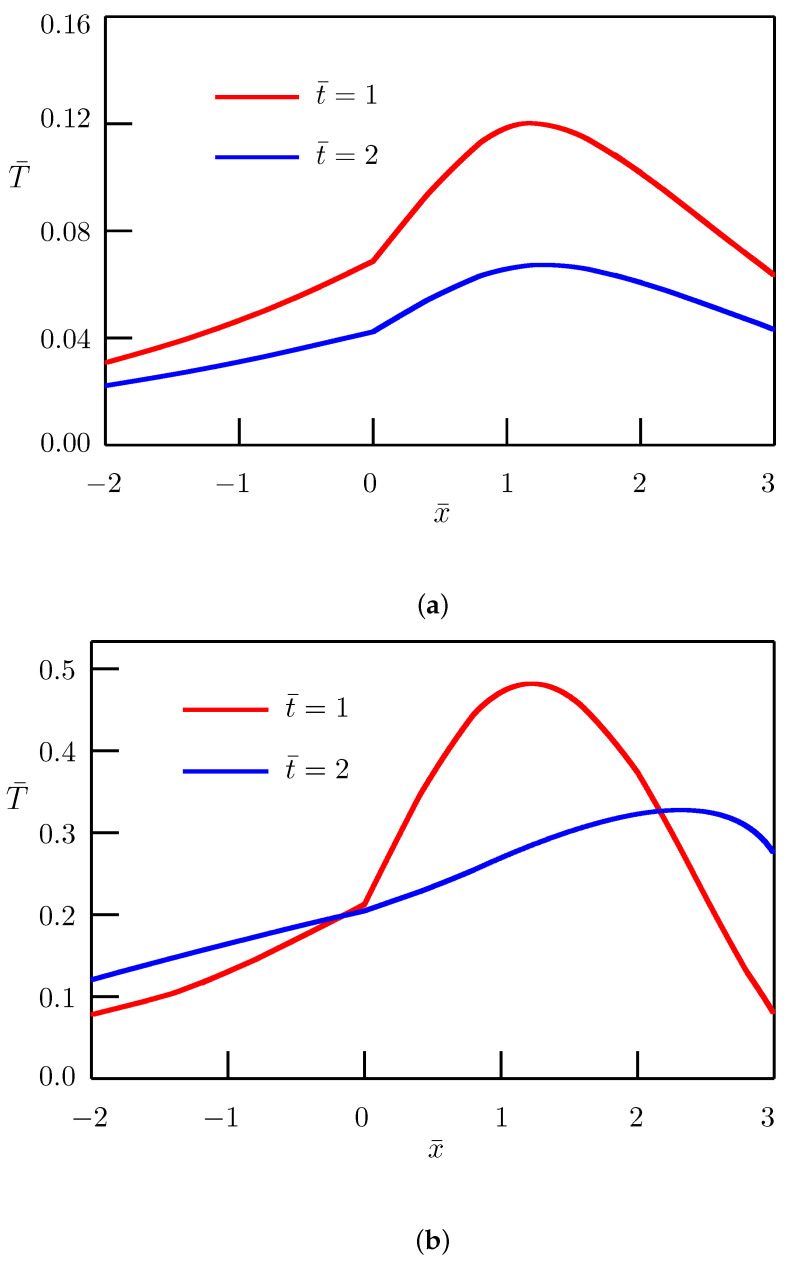
Fundamental solution to the source problem. Dependence of temperature on distance for α=0.5—(**a**), α=1.5—(**b**).

**Figure 3:**
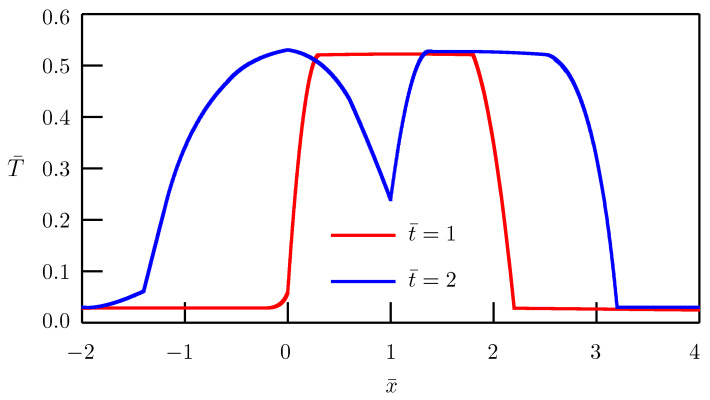
Fundamental solution to the source problem. Dependence of temperature on distance for α=1.98.

The dependence of the solution on distance is depicted in Figure 2 and Figure 3 for different values of the order of fractional derivative. In numerical simulations, we have utilized the nondimensional temperatures

(61) $${\bar{T}}_{1}=\frac{a_{1}^{1-1/\alpha }}{l^{1-2/\alpha }q_{0}}\;T_{1},\;{\bar{T}}_{2}=\frac{a_{1}^{1-1/\alpha }}{l^{1-2/\alpha }q_{0}}\;T_{2}.$$

Other nondimensional quantities are the same as in Equation (46).

## 4. Concluding Remarks

We have studied the fundamental solutions to the Cauchy problem along with the source problem for time-fractional heat conduction equations in a composite solid under conditions of nonperfect thermal contact. For an arbitrary function describing the initial condition or the source term, the solution can be obtained as a convolution of the corresponding fundamental solution and this function. It should be emphasized that, in the case of classical heat conduction (α=1), the fundamental solutions to the Cauchy problem and to the source problem coincide, whereas, in the case of fractional heat conduction, they are essentially different.

The fundamental solutions were obtained in terms of the generalized Mittag-Leffler, Mainardi, and Wright functions. In numerical simulations, we used the algorithms [20,21] for the calculation of these functions.

For the order of time-fractional derivative 0<α<1, the fractional heat conduction equation interpolates between the elliptic-type Helmholtz equation and the standard heat conduction equation of the parabolic type. When the order of time-fractional derivative 1<α<2, the fractional heat conduction equation interpolates between the classical heat conduction equation and the hyperbolic wave equation. In the instant of ballistic heat conduction (α=2), the wave fronts appear at $x=l+\sqrt{a_{1}}t$ and $x=l-\sqrt{a_{1}}t$ for x>0 and at $\left|x\right|=\sqrt{a_{1}}t-l\sqrt{a_{2}}/\sqrt{a_{1}}$, where $\sqrt{a_{1}}$ and $\sqrt{a_{2}}$ can be interpreted as speeds of heat wave propagation in contacting solids. Figure 3 shows how these wave fronts are approximated when the order of fractional derivative α approaches 2.

## Data Availability

Data are contained within the article.

## Appendix A. Fractional Integrals and Derivatives

Integral and differential operators of non-integer order are defined as follows [17,19]: the Riemann–Liouville fractional integral:

(A1) $$I^{\alpha }f\left(t\right)=\frac{1}{\Gamma (\alpha )}\int_{0}^{\;t}{\left(t-\tau \right)}^{\alpha -1}f\left(\tau \right)\;d\tau ,\;\alpha >0,\;$$

the Riemann–Liouville fractional derivative:

(A2) $$D_{RL}^{\alpha }f\left(t\right)=\frac{d^{n}}{dt^{n}}\left[\frac{1}{\Gamma (n-\alpha )}\int_{0}^{\;t}{\left(t-\tau \right)}^{n-\alpha -1}f\left(\tau \right)\;d\tau \right],\;n-1<\alpha <n,\;$$

the Caputo fractional derivative:

(A3) $$\frac{d^{\alpha }f\left(t\right)}{dt^{\alpha }}=\frac{1}{\Gamma (n-\alpha )}\int_{0}^{\;t}{\left(t-\tau \right)}^{n-\alpha -1}\frac{d^{n}f\left(\tau \right)}{d\tau^{n}}\;d\tau ,\;n-1<\alpha <n\;.\;$$

Recall the Laplace transform rules for fractional integrals and derivatives:

(A4) $$L\left\{I^{\alpha }f\left(t\right)\right\}=\frac{1}{s^{\alpha }}f^{*}\left(s\right),\;\alpha >0,\;$$

(A5) $$L\left\{D_{RL}^{\alpha }f\left(t\right)\right\}=s^{\alpha }f^{*}\left(s\right)-\sum_{k=0}^{n-1}\frac{\;d^{k}}{dt^{k}}I^{n-\alpha }f\left(0^{+}\right)\;s^{n-1-k},\;\;n-1<\alpha <n,\;$$

(A6) $$L\left\{\frac{d^{\alpha }f\left(t\right)}{dt^{\alpha }}\right\}=s^{\alpha }f^{*}\left(s\right)-\sum_{k=0}^{n-1}f^{\left(k\right)}\left(0^{+}\right)s^{\alpha -1-k},\;n-1<\alpha <n.\;$$

Here, the asterisk denotes the transform and *s* is the Laplace transform variable.

## References

[1] Tarasov V.E. (2010). Fractional Dynamics: Applications of Fractional Calculus to Dynamics of Particles, Fields and Media.

[2] Uchaikin V.V. (2013). Fractional Derivatives for Physicists and Engineers.

[3] Atanacković T.M., Pilipović S., Stanković B., Zorica D. (2014). Fractional Calculus with Applications in Mechanics: Vibrations and Diffusion Processes.

[4] Tarasov V.E. (2016). Heat transfer in fractal materials. Int. J. Heat Mass Transf..

[5] Pagnini G. (2019). Fractional kinetics in random/complex media. Handbook of Fractional Calculus with Applications, Volume 5: Applications in Physics, Part B.

[6] Zhmakin A.I. (2021). Heat conduction beyond the Fourier law. Techn. Phys..

[7] Zhmakin A.I. (2023). Non-Fourier Heat Conduction: From Phase-Lag Models to Relativistic and Quantum Transport.

[8] Zhou Y. (2024). Fractional Diffusion and Wave Equations: Well-Posedness and Inverse Problems.

[9] Povstenko Y. (2024). Fractional Thermoelasticity.

[10] Kharrat M., Touafek N., Krichen M. (2025). Modeling of Discrete and Continuous Systems: Ordinary, Partial and Fractional Derivatives.

[11] Povstenko Y., Kyrylych T. (2019). Fractional heat conduction in solids connected by thin intermediate layer: Nonperfect thermal contact. Continuum Mech. Thermodyn..

[12] Podstrigach Y.S. (1963). Temperature field in a system of solids conjugated by a thin intermediate layer. Inzh. Fiz. Zhurn..

[13] Povstenko Y. (2013). Fractional heat conduction in infinite one-dimensional composite medium. J. Therm. Stress..

[14] Prudnikov A.P., Brychkov Y.A., Marichev O.I. (1986). Integrals and Series, Volume 1: Elementary Functions.

[15] Mainardi F. (1996). The fundamental solutions for the fractional diffusion-wave equation. Appl. Math. Lett..

[16] Mainardi F. (1996). Fractional relaxation-oscillation and fractional diffusion-wave phenomena. Chaos Solitons Fractals.

[17] Podlubny I. (1999). Fractional Differential Equations.

[18] Gorenflo R., Kilbas A.A., Mainardi F., Rogosin S.V. (2020). Mittag-Leffler Functions, Related Topics and Applications.

[19] Kilbas A.A., Srivastava H.M., Trujillo J.J. (2006). Theory and Applications of Fractional Differential Equations.

[20] Gorenflo R., Loutchko J., Luchko Y. (2002). Computation of the Mittag-Leffler function *E*_α,β_(*z*) and its derivatives. Fract. Calc. Appl. Anal..

[21] Luchko Y. (2008). Algorithms for evaluation of the Wright function for the real arguments’ values. Fract. Calc. Appl. Anal..

